# 

**Published:** 1898-06-11

**Authors:** 


**Notices of Births, Deaths, and Marriages** are inserted in this column at a charge of 2s. 6d. for an announcement not exceeding four lines.

### NOTICE TO CORRESPONDENTS.

**All MS.,** Letters, Books for Review, and other matters intended for the Editor should be addressed The Editor, "The Hospital," The Hospital Building, 28 & 29, Southampton Street, Strand, W.C.

**The Editor** cannot undertake to return rejected MS., even when accompanied by stamped directed envelope.

**Notice to Advertisers and Subscribers.**

All Advertisements, Orders for Copies of The Hospital, and Business Communications should be addressed to **THE MANAGER, (Not to The Editor),** The Hospital, The Hospital Building, 28 & 29, Southampton Street, Strand, London, W.C.

The Cost of Subscription, post free, is as follows:—Per week, 2½d. per quarter, 2s. 8d.; per half-year, 5s. 3d.; per annum, 10s. 6d. Foreign:—Per annum, 15s. 2d.; payable, in all cases, in advance.

**NOTICE.—Vol. XXIII. of "The Hospital" (October, 1897, to March, 1898), in handsome cloth binding, is now on sale at the Office of this Journal, price 7s. 6d. Cases for binding the Numbers contained in this Volume, 1s. 6d. Index to Vol. XXIII., 2½d., post free.**

**The Monthly Part for June is now ready. Price 6d., by post 8d.**

### BOOKS RECEIVED.

Eyre and Spottiswoode.

"Board of Agriculture: Annual Report for 1897 under the Diseases of Animals Act, &c."

William Hodge, Glasgow.

"Annual Reports to County Councils of Dumbarton and of Stirling." By John C. McVail, M.D.

Cassell and Co.

"Tropical Diseases." By Patrick Manson, M.D., LL.D.

Bradley T. Batsford.

"The Building of Workhouses." By George H. Bibby, F.R.I.B.A.

Kegan Paul, Trench, Trubner, and Co.

"The Light of the West." By J. A. Goodchild.

"B. Bradshaw's Dictionary of Health Resorts."

The Women's Total Abstinence Union.

"Medicated Wines and Their Dangers." By Walter N. Edwards, F.C.S.

Robert Anderson, Glasgow.

"Descriptive Report on the Middle Ward Hospital (near Motherwell)." By Alex. Cullen, I A.

The Scientific Press.

"The Schott Treatment for Chronic Heart Disease." By Richard Greene, F.R.C.P.Ed. Second Edition.

"Pharmaceutical Journal" Office.

"Synopsis of the British Pharmacopœia (1898)."

The Werner Company.

"The Determination of Sex." By Dr. Leopold Schenk.

H. K. Lewis.

"Diseases of the Kidneys and Genito-Urinary Organs." Vol. II. By Professor Paul Fürbringer. Translated by W. H. Gilbert, M.D.

Frank A. Rogers.

"Important Alterations in the B.P. (1898)".

### Scraps and Gleanings.

Her Majesty the Queen, who is the patron of the Royal Hospital for Diseases of the Chest, in the City Road, contributed £25 to the Festival Dinner Fund.

A favourite first-aid treatment for diphtheria among the fishermen of Labrador—and the disease is rather prevalent there—is to split open a red herring and apply it to the affected throat.

The Mazawattee Tea Company have paid what is said to be the biggest cheque for customs duty of the age. It amounts to £63,147 2s. 10d., for 1,687 tons of tea, sufficient to brew one thousand million cups of tea.

The North London Hospital for Consumption has received donations of £25 from the Goldsmiths' Company and 20 guineas from the Skinners' Company.

An excellent entertainment was recently given by Mr. Charles Conway to the patients and nurses of the Royal Orthopædic Hospital. The programme consisted of recitations, songs, impersonations of some of Dickens's best-known characters, and a farce, entitled "Lydia's Lover's Lodgings."

Second Edition. Price 6d.,
THE SCHOTT TREATMENT
FOR
CHRONIC HEART DISEASES.
By RICHARD GREENE, F.R.C.P.Ed.

Crown 8vo., Cloth Gilt, with many Illustrations. Price 2/6,
SPINAL CURVATURE
AND
AWKWARD DEPORTMENT:
THEIR CAUSES AND PREVENTION IN CHILDREN.
By Dr. GEORGE MÜLLER,
Professor of Medicine and Orthopædics, Berlin.
English Edition. Edited and Adapted
By RICHARD GREENE, F.R.C.P.Ed.

London: The Scientific Press, Ltd., 28 & 29, Southampton Street, Strand, W.C.

